# Phenotypic age acceleration and early-onset lung cancer: a case–control and prognostic cohort study involving multiple clinical centres with validation in the UK Biobank

**DOI:** 10.1016/j.ebiom.2026.106162

**Published:** 2026-02-10

**Authors:** Ruolin Gao, Yuxuan Liao, Qiwen Zheng, Zhijian Xu, Xiaowei Zhao, Qinxiang Guo, Zhijie Wang, Jianchun Duan, Rui Wan, Jiachen Xu, Kailun Fei, Boyang Sun, Wei Zhuang, Hua Bai, Yanhui Cheng, Jia Zhong, Jie Wang

**Affiliations:** a State Key Laboratory of Molecular Oncology, CAMS Key Laboratory of Translational Research on Lung Cancer, Department of Medical Oncology, National Cancer Center/National Clinical Research Center for Cancer/Cancer Hospital, Chinese Academy of Medical Sciences and Peking Union Medical College, Beijing, 100021, China; b China National Center for Bioinformation, Beijing, 100101, China; c Beijing Institute of Genomics, Chinese Academy of Sciences, Beijing, 100101, China; d Department of Cancer Prevention, National Cancer Center/National Clinical Research Center for Cancer/Cancer Hospital, Chinese Academy of Medical Sciences and Peking Union Medical College, Beijing, 100021, China; e Department of Respiratory Medicine, Shanxi Province Cancer Hospital/ Shanxi Hospital Affiliated to Cancer Hospital, Chinese Academy of Medical Sciences/Cancer Hospital Affiliated to Shanxi Medical University, Taiyuan, Shanxi, 030013, China; f The First Affiliated Hospital of Henan University, Kaifeng, Henan, 475000, China

**Keywords:** Early-onset lung cancer, Biological ageing, Phenotypic age, Cancer risk, Prognosis

## Abstract

**Background:**

Lung cancer is mainly diagnosed in elderly adults, yet the incidence of early-onset disease in people aged ≤45 years is increasing. Current screening strategies largely target elder populations, leaving younger individuals at risk of delayed diagnosis and adverse outcomes. Phenotypic Age Acceleration (PhenoAgeAccel), which indicates the difference between biological age and chronological age, has been associated with cancer susceptibility, but its role in early-onset lung cancer is unclear.

**Methods:**

We performed a case–control and prognostic cohort study in China, including 222 early-onset lung cancer patients and 222 age- and sex-matched healthy volunteers, and externally validated findings in the UK Biobank. PhenoAgeAccel was calculated from routinely available haematological and biochemical markers. Logistic regression models estimated associations between PhenoAgeAccel and lung cancer risk. Survival analyses assessed the relationship between PhenoAgeAccel and overall survival among early-onset patients.

**Findings:**

Early-onset lung cancer patients had substantially higher PhenoAgeAccel than matched controls (*P* < 0.001). PhenoAgeAccel was associated with a dose-dependent increase in early-onset lung cancer risk (odds ratio [OR] = 1.18; 95% CI: 1.14–1.23). Subgroup analyses by chronological age demonstrated a stronger association in earlier adulthood, whereas associations among elder adults (≥65 years) were not significant. In early-onset patients, higher PhenoAgeAccel predicted worse overall survival (hazard ratio [HR] = 2.17; 95% CI: 1.20–3.93). Results were corroborated in the UK Biobank cohort.

**Interpretation:**

PhenoAgeAccel is positively associated with both a greater risk of early-onset lung cancer and poorer prognosis, supporting its potential utility for early detection and risk stratification in younger populations.

**Funding:**

10.13039/501100001809National Natural Science Foundation of China (no. 82303969); 10.13039/100018904Beijing Xisike Clinical Oncology Research Foundation (no. Y-2024AZ [EGFR]MS-0079); Beijing Natural Science Foundation (no. 7222144).

**Research in context**

**Evidence before this study**

We searched PubMed using the terms “(young OR early onset) AND (lung cancer OR lung carcinoma OR carcinoma of lung)” and “(lung cancer OR lung carcinoma OR carcinoma of lung) AND (ageing OR senescence OR phenotypic age)”, with no date or language restrictions. Prior work on early-onset lung cancer has concentrated on its biological and clinical characteristics, but has often been limited by small samples. Studies examining biological ageing and cancer risk have produced conflicting results, and there is a paucity of research specifically linking biological ageing to early-onset lung cancer or examining its prognostic significance.

**Added value of this study**

Using a case–control and prognostic cohort study design involving multiple clinical centres with independent validation in the longitudinal UK Biobank, this study provides robust evidence that Phenotypic Age Acceleration (PhenoAgeAccel) is elevated in patients with early-onset lung cancer and is associated with a dose-dependent increase in disease risk. The association persists after accounting for conventional risk factors, and we show that PhenoAgeAccel strongly predicts poorer survival in early-onset patients.

**Implications of all the available evidence**

Contemporary lung cancer screening eligibility focuses on chronological age and smoking history. Our findings suggest that incorporating biological ageing metrics, such as PhenoAgeAccel, could enhance the identification of younger individuals at elevated risk who current criteria would otherwise overlook. Integrating PhenoAgeAccel into clinical risk stratification, developing ageing-targeted prevention strategies, and re-evaluating screening policies could enhance early detection and ultimately improve outcomes for early-onset lung cancer patients.

## Introduction

Lung cancer remains the leading cause of cancer mortality worldwide and was the most frequently diagnosed cancer in 2022, accounting for approximately 2.5 million new cases (12.4% of all cancers) and 1.8 million deaths (18.7% of all cancer deaths).^1^ Although lung cancer is generally considered an age-related disease with a median age at diagnosis of about 70 years, a distinct subset of early-onset lung cancer in younger adults (commonly defined as <40–50 years) is increasingly recognised and represents an important public-health concern.^2^, ^3^, ^4^ In 2019, the global age-standardised incidence, mortality, and disability-adjusted life-year rates for early-onset lung cancer were reported as 2.82, 2.28, and 106.47 per 100,000 person-years, respectively.^5^ The burden is concentrated in low- and middle-sociodemographic index countries, where rapid economic development, rising smoking prevalence, westernised lifestyles, and environmental pollution are likely contributing factors.^6^

Early-onset lung cancer differs from later-onset disease in several clinical and biological respects: demographic patterns (sex distribution and smoking status), comorbidity profiles, and distinct molecular features.^7^, ^8^, ^9^, ^10^ At the genomic level, younger patients show higher frequencies of actionable driver alterations (for example, *ALK* and *ROS1* fusions), a higher rate of germline variants, and, paradoxically, lower tumour mutational burden (TMB), reduced genomic heterogeneity, and simpler tumour evolutionary trajectories.^11^, ^12^, ^13^ Whether early-onset disease carries a better or worse prognosis than late-onset disease remains contested, with outcomes closely tied to stage at diagnosis.^2^^,^^9^^,^^14^, ^15^, ^16^ Earlier detection is therefore crucial, yet widely accessible, cost-effective screening methods for younger populations are lacking.^17^ Although bodies such as the American Cancer Society recommend annual low-dose computed tomography (LDCT) for asymptomatic individuals aged 50–80 years, current guidelines do not capture many younger at-risk individuals.^10^^,^^18^

Ageing is a complex, multisystem process that progressively diminishes physiological reserve and heightens vulnerability to chronic disease, including cancer.^19^ Phenotypic age (PhenoAge), derived from nine clinical chemistry biomarkers, is a validated surrogate that often better reflects biological rather than chronological ageing.^20^, ^21^, ^22^ Studies investigating PhenoAge and cancer risk have produced heterogeneous findings: several reports describe clear links between PhenoAge and cancer susceptibility, including lung cancer,^23^, ^24^, ^25^ whereas others do not find a significant association.^26^^,^^27^ Evidence on whether biological ageing influences cancer prognosis is sparse.

In this study, we designed two studies to examine whether Phenotypic Age Acceleration (PhenoAgeAccel), estimated from routine haematological and biochemical tests, is associated with the risk of early-onset lung cancer and with prognosis among affected patients. One was a case–control study involving multiple clinical centres in China with external validation in the UK Biobank cohort was used to evaluate the relationship between PhenoAgeAccel and early-onset lung cancer risk. The other was a following prognostic cohort study to assess the prognostic value of PhenoAgeAccel in early-onset lung cancer patients. Our goal was to evaluate PhenoAgeAccel as a simple, feasible screening and prognostic indicator that could be derived from routine clinical data to aid risk stratification and inform future assessments of clinical utility.

## Methods

### Discovery sample in the case–control and prognostic cohort study

**Fig. 1:**
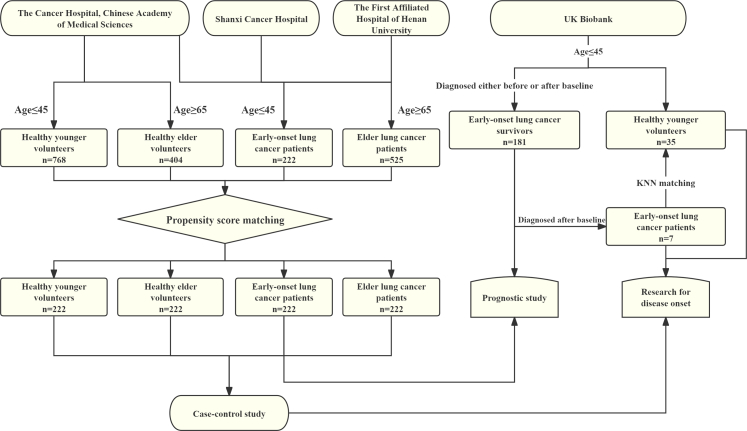
**Flowchart of the study**.

The study design is illustrated in Fig. 1, with sample size calculations detailed in the Supplementary Material. Patient inclusion criteria were: (1) histologically or cytologically confirmed lung cancer diagnosed between 2016 and 2022 at the Cancer Hospital, Chinese Academy of Medical Sciences, Shanxi Cancer Hospital, or The First Affiliated Hospital of Henan University; (2) age at diagnosis ≤45 years (younger lung cancer) or ≥65 years (elder lung cancer); (3) no prior anticancer treatment; and (4) complete clinical and demographic data available. In total, 1278 younger and 2890 elder lung cancer patients were identified.

Exclusion criteria were: (1) insufficient blood sample data for PhenoAge calculation; (2) loss to follow-up; (3) acute infection at the time of blood collection; and (4) diagnosis not being the first primary lung cancer at presentation. After excluding 1056 patients, 222 younger patients with lung cancer were included: 180 from the Cancer Hospital, Chinese Academy of Medical Sciences, 20 from Shanxi Cancer Hospital, and 22 from The First Affiliated Hospital of Henan University. Similarly, after excluding 2365 patients, 525 elderly lung cancer patients were included: 444 from the Cancer Hospital, Chinese Academy of Medical Sciences; 47 from Shanxi Cancer Hospital; and 34 from The First Affiliated Hospital of Henan University. Full inclusion and exclusion procedures are provided in Figure S1.

PhenoAge was calculated using blood samples collected at first diagnosis. Propensity score matching (PSM) was applied to minimise potential confounding between groups. For matching younger and elder lung cancer patients, propensity scores were derived via logistic regression incorporating sex, smoking history, family history, histology, ECOG performance status, TNM stage (T, N, M classification), tumour laterality, and treatment centre as covariates (Table S1). Matching was performed using a 1:1 nearest-neighbour algorithm with a calliper width of 0.2 standard deviations of the logit of the propensity score, yielding 222 elder lung cancer patients matched to the 222 younger patients. Covariate balance was assessed using standardised mean differences (SMD).

Healthy volunteers were recruited to represent the general cancer-free population. Inclusion criteria were: (1) participation in health examinations at the Department of Cancer Prevention, Cancer Hospital, between 2016 and 2022; (2) regular cancer screening follow-up; (3) age ≤45 years (younger) or ≥65 years (elder) at examination; and (4) complete demographic information available. Initially, 3229 younger and 910 elder healthy volunteers were identified. Exclusion criteria were: (1) insufficient blood sample data for PhenoAge calculation; (2) acute infection at blood collection; and (3) development of any malignancy during follow-up (by 15 November 2024). After excluding 2461 younger and 506 elder volunteers, 768 younger and 404 elder healthy volunteers remained eligible (Figure S1). Using the same PSM method, 222 younger and 222 elder healthy volunteers were matched to their respective case groups by age, sex, smoking history, and family history (Tables S2 and S3).

### Follow-up of early-onset lung cancer patients in the prognostic cohort study

In the prognostic cohort study, early-onset lung cancer patients were followed up regularly for survival. Overall survival (OS) was defined as the time from diagnosis to death or last follow-up. The final follow-up date was 15 November 2024.

### Validation using the UK Biobank

The UK Biobank is a large-scale, ongoing prospective study of ∼500,000 participants aged 37–73 years at recruitment (2006–2010) in 22 assessment centres from England, Scotland, and Wales. All participants completed baseline physical assessments, questionnaires, and provided blood samples for blood routine and biochemical assays used to calculate PhenoAge and PhenoAgeAccel.^28^ For validation, we included 181 lung cancer survivors diagnosed before age 45 with complete data and a median follow-up of 121 months for prognostic study. Among these, seven were cancer-free at baseline but developed lung cancer during follow-up; these were included in analyses of PhenoAgeAccel and early-onset lung cancer risk.

Healthy volunteers were defined as UK Biobank participants with no cancer record, no self-reported prior malignancy, and, for deceased individuals, no cancer listed as the cause of death. In view of severe case–control imbalance, matching of controls to cases was performed using the k-nearest neighbour method.^29^ Continuous variables (*e.g*., age) were standardised, and categorical variables (*e.g*., sex, race, smoking status) were one-hot encoded. Euclidean distances in the resulting multidimensional feature space were calculated, and for each early-onset lung cancer patient, the five nearest healthy controls were selected (1:5 ratio) without replacement. This approach balanced statistical power with matching quality. The process yielded seven early-onset lung cancer cases and 35 matched young healthy volunteers. Given the large participant pool and consistent recruitment period (2006–2010), follow-up duration was broadly comparable across individuals and was therefore not included as a matching variable.

### PhenoAge and PhenoAgeAccel

PhenoAge was calculated according to previously described methods, with further details provided in the Supplementary Material.^30^ PhenoAgeAccel was defined as the residual from a linear regression of PhenoAge on chronological age, reflecting whether an individual appears biologically elder (positive values) or younger (negative values) than their chronological age.^31^ In the onset study, PhenoAgeAccel was first treated as a continuous variable and then analysed as an ordinal variable categorised into quartiles (Q1–Q4) for interpretability.

### Statistical analyses

All analyses were conducted using R version 4.2.0 (The R Foundation for Statistical Computing) and SPSS version 24.0 (IBM Corp., Armonk, NY, USA). Detailed sample size calculation and power analysis was provided in the Supplementary Material. Normality was assessed with the Kolmogorov–Smirnov test. Continuous variables are presented as mean ± standard deviation (SD) or median and interquartile range (IQR), depending on distribution. Between-group comparisons of continuous variables used either the Wilcoxon rank-sum test or the Welch two-sample *t*-test, according to data characteristics. For categorical variables, the Fisher exact test was applied where expected frequencies were <5; otherwise, the chi-squared test was used. Differences in the overall distribution of PhenoAgeAccel between healthy individuals and patients with lung cancer were evaluated with the Kolmogorov–Smirnov test.

For the case–control study in the relationship between PhenoAgeAccel and lung cancer risk was estimated using separate logistic regression models, and the reported odds ratios (ORs) represent unadjusted associations with early-onset lung cancer risk. Univariable and multivariable logistic regression models examined associations between nine PhenoAge-based biomarkers and lung cancer risk, stratified by chronological age group (younger vs. elder). Interaction terms assessed whether associations varied by chronological age, sex, or cancer stage. Receiver operating characteristic (ROC) curve analysis evaluated the predictive performance of PhenoAgeAccel.

For the prognosis cohort study in younger lung cancer patients, Kaplan–Meier plots illustrated overall survival (OS) differences between low and high PhenoAgeAccel groups, and Cox proportional hazards regression analysis was applied. PhenoAgeAccel was dichotomised using a data-driven cutoff derived from Cox proportional hazards modelling. Briefly, all observed values of PhenoAgeAccel were scanned as candidate thresholds, and for each potential split, a Cox model was fitted to evaluate its association with overall survival. The threshold corresponding to the most statistically significant model fit (based on the Wald test for the group variable) was selected as the final cutoff for defining low and high PhenoAgeAccel groups. The multivariable model adjusted for relevant clinical and demographic covariates—age, sex, smoking history, family history, histology, stage, ECOG performance status, mutation status, previous treatments, and study centre for the case–control cohort; age, sex, smoking history, family history, race, Townsend deprivation index, and BMI for the UK Biobank—to minimise confounding and ensure covariate balance. Additional subgroup analyses based on mutation status (EGFR mutation, non-EGFR mutation, or wild type) were conducted using univariable Cox regression. *P* < 0.05 was considered statistically significant.

### Ethics

The case–control and prognostic cohort study were conducted in accordance with the 2024 Declaration of Helsinki ethical principles, and were approved by the Ethics Committee of the Cancer Hospital, Chinese Academy of Medical Sciences (no. NCC2024C-1077). Written informed consent was obtained from all participants. UK Biobank data were accessed under Application no. 104784, and all procedures followed UK Biobank Ethics and Governance Framework.

### Role of funders

The funding bodies had no involvement in study design, data collection, statistical analysis, data interpretation, or manuscript preparation.

## Results

### Participant characteristics and PhenoAge metrics in the case–control study

Table 1 summarises the clinical characteristics and PhenoAge values of lung cancer patients and healthy volunteers in the case–control cohort. Among younger participants, the mean PhenoAge was significantly higher in lung cancer patients than in matched healthy controls (38.4 ± 7.39 vs. 32.3 ± 6.73 years; *P* < 0.001). Similarly, PhenoAgeAccel was markedly higher in younger patients (−0.88 ± 6.47 vs. −5.75 ± 4.55 years; *P* < 0.001), whereas chronological age did not differ significantly (*P* = 1).

**Table 1:** Characteristics of the patients with lung cancer and healthy volunteers in the case–control study.

Variable	Younger adults with lung cancer N = 222	Healthy younger volunteers N = 222	*P*-value	Elder adults with lung cancer N = 222	Healthy elder volunteers N = 222	*P*-value
Age						
PhenoAge, years	38.4 ± 7.39	32.3 ± 6.73	**<0.001**	67.0 ± 7.47	65.9 ± 6.52	0.086
PhenoAgeAccel, years	−0.88 ± 6.47	−5.75 ± 4.55	**<0.001**	−1.95 ± 6.34	−3.04 ± 6.59	0.075
Chronological age, years	40 ± 5.15	40 ± 5.10	1	68 ± 3.92	69 ± 3.34	0.886
Sex						
Male	102 (45.9)	107 (48.2)	1	114 (51.4)	117 (52.7)	0.849
Female	120 (54.1)	115 (51.8)		108 (48.6)	105 (47.3)	
Smoking history						
No	145 (65.3)	144 (64.5)	0.909	133 (59.9)	144 (64.9)	0.291
Yes	77 (34.7)	78 (35.5)		89 (40.1)	78 (35.1)	
Family history						
No	162 (73.0)	159 (71.6)	0.747	163 (73.4)	160 (72.1)	0.748
Yes	60 (27.0)	63 (28.4)		59 (26.6)	62 (27.9)	
Albumin, g/L	44.40 ± 5.81	46.10 ± 2.34	**<0.001**	43.30 ± 3.27	43.65 ± 3.23	0.153
Alkaline phosphatase, U/L	78.00 ± 46.54	61.75 ± 21.43	**<0.001**	82.55 ± 34.22	74.2 ± 19.81	**<0.001**
Creatinine, umol/L	67.9 ± 11.63	64.00 ± 14.25	0.072	69.1 ± 15.32	69.0 ± 15.42	0.543
Glucose, mmol/L	5.24 ± 1.35	5.03 ± 0.79	**<0.001**	5.69 ± 5.67	5.60 ± 1.15	0.070
C-reactive protein, mg/dL	0.24 ± 1.55	0.04 ± 0.19	**<0.001**	0.40 ± 4.41	0.1 ± 0.36	**<0.001**
Mean cell volume, fL	89.00 ± 13.87	89.95 ± 9.31	**<0.001**	92.0 ± 4.04	92.2 ± 4.05	0.177
Red cell distribution width, n (%)	12.5 ± 8.95	12.30 ± 5.28	**0.018**	12.7 + 5.33	12.6 ± 8.14	0.119
White blood cell count, 1000 cells/uL	7.17 ± 2.33	5.64 ± 7.94	**<0.001**	6.89 ± 2.29	5.72 ± 1.41	**<0.001**
Lymphocyte percentage, n (%)	24.1 ± 9.09	32.95 ± 7.88	**<0.001**	27.55 ± 7.68	30.2 ± 7.85	0.072

PhenoAgeAccel was defined as the residual from a linear regression model of PhenoAge on chronological age. Data were presented as the mean ± standard deviation (SD) for continuous variables and as n (%) for categorical variables. *P-*values < 0.05 were bolded, indicating statistical significance.

The comparison of continuous variables between groups was conducted using the Wilcoxon rank-sum test or the Welch two-sample *t* test, depending on the data characteristics. Chi-squared test was used for the statistical analysis of categorical variables. PhenoAge acceleration, PhenoAgeAccel.

Compared with healthy peers, younger lung cancer patients showed significantly lower serum albumin (44.40 ± 5.81 vs. 46.10 ± 2.34 g/L; *P* < 0.001) and lymphocyte percentages (*P* < 0.001), alongside higher glucose (5.24 ± 1.35 vs. 5.03 ± 0.79 mmol/L; *P* < 0.001), alkaline phosphatase (78.00 ± 46.54 vs. 61.75 ± 21.43 U/L; *P* < 0.001), C-reactive protein (CRP) (0.24 ± 1.55 vs. 0.04 ± 0.19; *P* < 0.001), and white blood cell (WBC) counts (7.17 ± 2.33 vs. 5.64 ± 7.94; *P* < 0.001). Among elder participants, differences in PhenoAge and PhenoAgeAccel were smaller and not statistically significant (*P* = 0.086 and *P* = 0.075, respectively).

### Positive association between PhenoAgeAccel and early-onset lung cancer risk

**Fig. 2:**
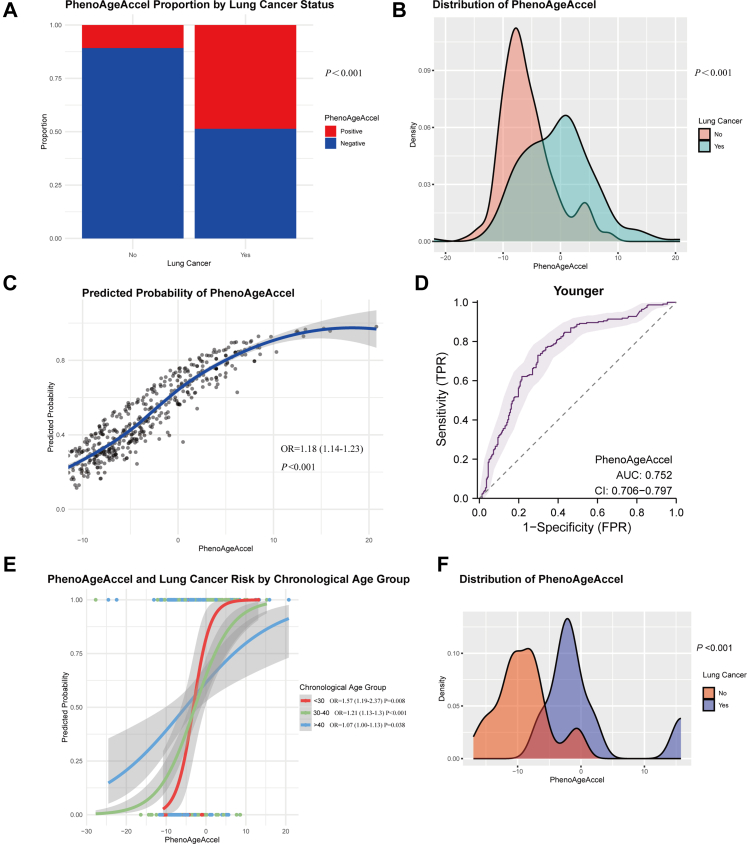
**Association between PhenoAgeAccel and early-onset lung cancer.** (A) Proportion of positive and negative phenotypic age acceleration (PhenoAgeAccel) in younger lung cancer patients and healthy volunteers, with a significantly higher ratio of positive PhenoAgeAccel in cancer cases (chi-squared test *P* < 0.001). (B) Density distribution illustrating the distribution of PhenoAgeAccel in younger lung cancer patients and healthy volunteers, showing a distinct rightward shift in the cancer patient group (Kolmogorov–Smirnov test, *P* < 0.001). (C) Scatterplot depicting the positive linear correlation between PhenoAgeAccel and lung cancer risk in younger adults (logistic regression, OR = 1.18, 95% CI: 1.14–1.23; *P* < 0.001). (D) Receiver operating characteristic (ROC) curve assessing the predictive performance of PhenoAgeAccel for early-onset lung cancer, yielding an area under the curve (AUC) of 0.75 (95% CI: 0.706–0.797). (E) Association between PhenoAgeAccel and lung cancer risk across different chronological age groups. (F) Density distribution of PhenoAgeAccel among younger lung cancer patients and matched healthy volunteers in the UK Biobank cohort, demonstrating a significant rightward shift in the cancer patient group (Kolmogorov–Smirnov test, *P* < 0.001).

Younger lung cancer patients had a significantly higher proportion of positive PhenoAgeAccel than healthy volunteers (Fig. 2A; *P* < 0.001). The distribution of PhenoAgeAccel was shifted towards higher values in younger patients (Fig. 2B; *P* < 0.001).

PhenoAgeAccel was positively associated with lung cancer risk in younger adults (per SD increase: 18% higher risk; 95% CI: 13.5–23.3%; *P* < 0.001; Fig. 2C; Table 2). Quartile analysis revealed a dose–response relationship: compared with the lowest quartile (Q1), the ORs for early-onset lung cancer were 1.58 (95% CI: 0.88–2.88; *P* = 0.131) for Q2, 4.86 (95% CI: 2.74–8.8; *P* < 0.001) for Q3, and 11.7 (95% CI: 6.32–22.65; *P* < 0.001) for Q4. The ROC analysis yielded an AUC of 0.752, indicating good discriminatory capacity (Fig. 2D).

**Table 2:** Associations between PhenoAgeAccel and early-onset lung cancer risk in younger adults in the case–control study.

	Odds ratio (OR)	95% confidence intervals (CI)	*P*-value
PhenoAgeAccel (continuous)	1.18	1.14–1.23	**<0.001**
PhenoAgeAccel (quartiles)			
Q1	Ref.		
Q2	1.58	0.88–2.88	0.131
Q3	4.86	2.74–8.80	**<0.001**
Q4	11.7	6.32–22.65	**<0.001**
Chronological age	1.05	1.01–1.10	**0.029**

PhenoAgeAccel was defined as the residual from a linear regression model of PhenoAge on chronological age.

Q1: −27.68 to −7.84 years; Q2: −7.84 to −4.32 years; Q3: −4.32 to 0.82 years; Q4: 0.82–20.77 years. *P-*values < 0.05 were bolded, indicating statistical significance.

Separate logistic regression was used to estimate the relationship between each PhenoAgeAccel related variable and lung cancer risk. The odds ratios (ORs) reported were unadjusted ORs, representing the association between each individual variable and early-onset lung cancer risk without adjusting for other covariates.

PhenoAgeAccel, PhenoAge acceleration.

**Fig. 3:**
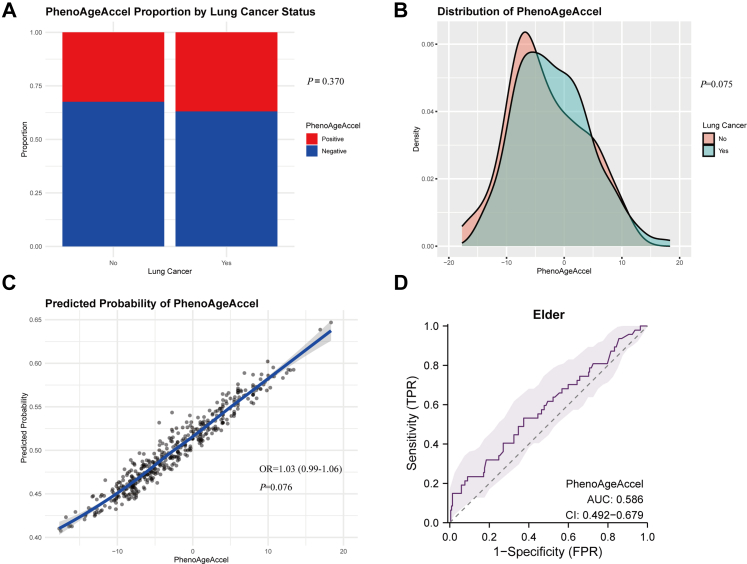
**Association of PhenoAgeAccel with cancer risk in elderly lung cancer patients.** (A) Proportion of positive and decelerated negative phenotypic age acceleration (PhenoAgeAccel) among elder lung cancer patients and age-matched healthy volunteers, showing no statistical significance (chi-squared test, *P* = 0.37). (B) Density distribution indicating no significant difference in the overall distribution of PhenoAgeAccel between the elder adult lung cancer patients and healthy volunteers (Kolmogorov–Smirnov test, *P* = 0.075). (C) Scatterplot showing a slight linear correlation between PhenoAgeAccel and lung cancer risk in elder adults with no statistical significance (logistic regression, OR = 1.03, 95% CI: 0.99–1.06; *P* = 0.076). (D) Receiver operating characteristic (ROC) curve assessing the predictive capacity of PhenoAgeAccel for lung cancer in elderly adult lung cancer patients, with an area under the curve (AUC) of 0.586 (95% CI: 0.492–0.679).

The effect of chronological age within the younger group was also examined. Regression slopes were steeper in younger adults, indicating a stronger impact of PhenoAgeAccel on lung cancer risk earlier in adulthood (Fig. 2E, Table 3). Among individuals ≤30 years, the OR per unit increase in PhenoAgeAccel was 1.57 (95% CI: 1.19–2.37; *P* = 0.008), compared with 1.07 (95% CI: 1.00–1.13; *P* = 0.038) for those aged ≥40 years. In the elder group (≥65 years), the association was weaker and non-significant (Fig. 3C, OR = 1.03, 95% CI: 0.99–1.06; *P* = 0.076). Elder lung cancer patients showed no significant differences in PhenoAgeAccel compared to age-matched controls (Fig. 3A; *P* = 0.37) and exhibited no significant distributional differences (Fig. 3B; *P* = 0.075). Predictive performance was poor in this group (AUC = 0.586; Fig. 3D).

**Table 3:** Associations between PhenoAgeAccel and lung cancer risk in chronological age, sex, and cancer stage subgroups.

Chronological age	Odds ratio (OR)	95% confidence intervals (CI)	*P-*value
Early-onset lung cancer (≤45 years)	1.18	1.14–1.23	**<0.001**
≤30 years	1.57	1.19–2.37	**0.008**
30–40 years	1.21	1.13–1.30	**<0.001**
40–45 years	1.07	1.00–1.13	**0.038**
Elder lung cancer (≥65 years)	1.03	0.99–1.06	0.076
Sex			
Male	1.16	1.10–1.23	**<0.001**
Female	1.19	1.13–1.27	**<0.001**
Stage			
I	1.03	0.95–1.12	0.421
II	1.15	1.03–1.31	**0.017**
III	1.18	1.12–1.25	**<0.001**
IV	1.23	1.17–1.30	**<0.001**

PhenoAgeAccel was defined as the residual from a linear regression model of PhenoAge on chronological age. *P-*values < 0.05 were bolded, indicating statistical significance.

Logistic regression models with interaction terms (PhenoAgeAccel × chronological age group) were applied to assess differences in associations across chronological age strata (≤30, 30–40, 40–45, and ≥65 years). Early-onset lung cancer was defined as ≤45 years at diagnosis, and elder lung cancer as ≥65 years. Subgroup analyses stratified by sex and cancer stage (I–IV) were also conducted in early-onset lung cancer with separate logistic regression models. The reported odds ratios represent associations within each subgroup and are unadjusted for other covariates.

PhenoAgeAccel, PhenoAge acceleration.

Stage-stratified analyses revealed progressively stronger effects of PhenoAgeAccel with advancing cancer stage (Figure S2A). A significant association was present even at stage II (OR = 1.15, 95% CI: 1.03–1.31; *P* = 0.017; Table 3). In early-stage disease (stages I–II), the AUC for discriminating cases from controls was 0.626 (95% CI: 0.515–0.737; Figure S2B).

Sex-stratified analyses revealed consistent positive associations in males (OR = 1.16, 95% CI: 1.10–1.23; *P* < 0.001) and females (OR = 1.19, 95% CI: 1.13–1.27; *P* < 0.001), with no evidence of interaction (*P* = 0.554; Figure S2C; Table 3). AUC values were 0.719 (95% CI: 0.653–0.785; Figure S2D) in males and 0.791 (95% CI: 0.726–0.855; Figure S2E) in females. Although slightly higher in females, predictive performance was broadly comparable across sexes, underscoring the robustness of PhenoAgeAccel as a risk indicator.

To validate the observed association, we analysed data from newly diagnosed early-onset lung cancer patients and matched healthy volunteers in the UK Biobank. Despite having comparable chronological ages, patients exhibited higher PhenoAge and PhenoAgeAccel values than their matched younger, healthy counterparts (Table 4; *P* < 0.001). Within the patient group, lower serum albumin (*P* = 0.017) and lymphocyte percentage (*P* = 0.040), together with higher CRP levels (*P* < 0.001), were observed—findings consistent with our case–control results. A density plot demonstrated a rightward shift in PhenoAgeAccel among patients relative to healthy individuals, reinforcing the potential link between PhenoAgeAccel and early-onset lung cancer risk (Fig. 2F; *P* < 0.001).

**Table 4:** Characteristics of the younger patients with early-onset lung cancer and healthy volunteers in the UK Biobank validation.

Variable	Younger adults with lung cancer N = 7	Healthy younger volunteers N = 35	*P*-value
Age			
PhenoAge, years	39.8 (38.5, 41.1)	33.1 (30.3, 34.9)	**<0.001**
PhenoAgeAccel, years	−1.5 (−3.0, 0.3)	−9.5 (−11.3, −7.4)	**<0.001**
Chronological age, years	42.00 (41.00, 42.50)	42.00 (41.00, 43.00)	1
Sex			
Male	2 (28.6%)	10 (28.6%)	1
Female	5 (71.4%)	25 (71.4%)	
Smoking history			
No	4 (57.1%)	20 (57.1%)	1
Yes	3 (42.9%)	15 (42.9%)	
Family history			
No	5 (71.4%)	25 (71.4%)	1
Yes	2 (28.6%)	10 (28.6%)	
Race			
Non-White	0 (0%)	0 (0%)	1
White	7 (100%)	35 (100%)	
Townsend	0.6 (−1.9, 1.6)	−3.3 (−4.1, 2.4)	0.280
BMI	25.9 ± 5.0	27.3 ± 4.6	0.510
Albumin, g/L	44.11 (42.66, 44.72)	46.75 (44.86, 48.38)	**0.017**
Alkaline phosphatase, U/L	91 (73, 107)	71 (61, 81)	0.200
Creatinine, umol/L	70 ± 17	76 ± 13	0.461
Glucose, mmol/L	4.80 (4.66, 4.94)	4.86 (4.67, 5.11)	0.500
C-reactive protein, mg/dL	0.54 (0.38, 2.69)	0.07 (0.04, 0.21)	**<0.001**
Mean cell volume, fL	90.2 ± 4.8	90.1 ± 3.0	0.939
Red cell distribution width, n (%)	13.90 (13.09, 14.29)	13.27 (12.87, 13.70)	0.182
White blood cell count, 1000 cells/uL	7.33 (6.33, 8.85)	6.60 (5.28, 8.09)	0.380
Lymphocyte percentage, n (%)	21 ± 6	28 ± 7	**0.040**

PhenoAgeAccel was defined as the residual from a linear regression model of PhenoAge on chronological age.

According to the distribution characteristics, continuous variables were presented as the mean ± standard deviation (SD) or as the median (IQR). Categorical variables were presented as n (%). *P-*values < 0.05 were bolded, indicating statistical significance.

The comparison of continuous variables between groups was conducted using the Wilcoxon rank-sum test or the Welch two-sample t test, depending on the data characteristics. For comparison between groups of categorical data, we used the Fisher exact test for expected frequencies of <5; otherwise, we used the chi-squared test.

BMI, body mass index; PhenoAgeAccel, PhenoAge acceleration.

### Associations between nine PhenoAge-related biomarkers and early-onset lung cancer risk

**Fig. 4:**
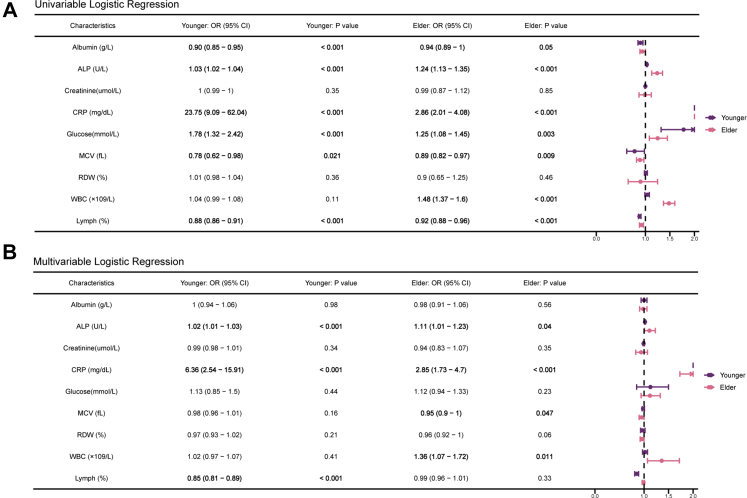
**Univariable and multivariable logistic regression analyses of PhenoAge biomarkers.** (A) Univariable logistic regression results for nine biomarkers in lung cancer risk stratified by age group. (B) Multivariable logistic regression results with simultaneous inclusion of all nine biomarkers in lung cancer risk stratified by age group. Odds ratios (ORs) and 95% confidence intervals (CIs) were plotted for each biomarker. Biomarkers with statistically significant associations (*P* < 0.05) were highlighted.

We next examined the associations between nine PhenoAge-related biomarkers and lung cancer risk using univariable and multivariable logistic regression analyses (Fig. 4). Although some biomarkers—such as alkaline phosphatase (ALP) and mean cell volume (MCV)—were statistically significant in univariable models, their ORs were close to 1.0 (*e.g*., ALP: OR = 1.03; MCV: OR = 0.92), indicating minimal effect sizes and warranting cautious interpretation. Accordingly, our emphasis was placed on multivariable models, which adjusted for potential confounders and provided more reliable effect estimates. Among younger individuals, CRP and lymphocyte percentage remained significantly associated with lung cancer risk after adjustment. CRP demonstrated a strong positive association (OR = 6.36, 95% CI: 2.54–15.91; *P* < 0.001), whereas lymphocyte percentage showed an inverse association (OR = 0.85, 95% CI: 0.81–0.89; *P* < 0.001). In contrast, corresponding effect sizes in the elder group were weaker.

### PhenoAgeAccel and prognosis in early-onset lung cancer

**Fig. 5:**
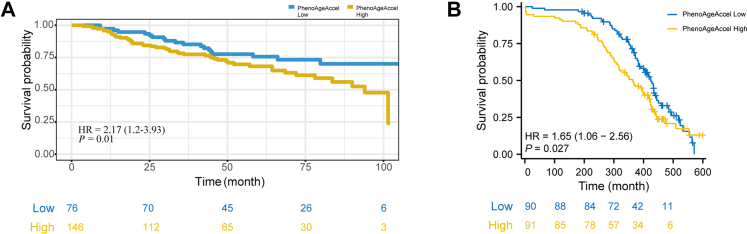
**PhenoAgeAccel and overall survival in early-onset lung cancer.** (A) Kaplan–Meier survival curves from the prognostic cohort study illustrating a significant difference in overall survival (OS) between early-onset lung cancer patients with high versus low phenotypic age acceleration (PhenoAgeAccel) in Chinese population. Patients in the high PhenoAgeAccel group exhibited significantly worse OS outcomes (multivariable Cox analysis, HR = 2.17, 95% CI: 1.2–3.93; *P* = 0.01), suggesting a strong link between PhenoAgeAccel and adverse prognosis. The high PhenoAgeAccel group was defined as those with a value greater than or equal to −3.42, while the low PhenoAgeAccel group included those with a value less than −3.42. A cutoff of −3.42 was determined based on the optimal *P*-value calculation. (B) Kaplan–Meier survival curves indicating that high PhenoAgeAccel is associated with poorer OS outcomes (multivariable Cox analysis, HR = 1.65, 95% CI: 1.06–2.56; *P* = 0.027) in UK Biobank validation. The high PhenoAgeAccel group was defined as those with a value greater than or equal to −6.05, while the low PhenoAgeAccel group included those with a value less than −6.05. A cutoff of −6.05 was determined based on the optimal *P*-value calculation. The overall survival time shown referred to the period from lung cancer diagnosis to death.

We further assessed the relationship between PhenoAgeAccel and prognosis in early-onset lung cancer by stratifying patients according to PhenoAgeAccel values and generating Kaplan–Meier survival curves. Data from our prognostic cohort (Fig. 5A; Table S4) showed that patients in the low-PhenoAgeAccel subgroup had significantly better OS than those in the high-PhenoAgeAccel subgroup (HR = 2.17, 95% CI: 1.20–3.93; *P* = 0.01), indicating that higher PhenoAgeAccel is associated with poorer prognosis in this population. To explore whether this prognostic effect persisted across molecular subtypes, we conducted stratified analyses based on mutation status, categorising patients as EGFR-mutant (n = 64), non-EGFR mutant (n = 33), or wild-type (WT; n = 61). Kaplan–Meier curves for each group (Figure S3) revealed that in the WT subgroup (Figure S3C), high PhenoAgeAccel was significantly linked to inferior OS (HR = 3.15, 95% CI: 1.00–15.80; *P* = 0.05). We also utilised a multivariable model that incorporated all relevant treatment variables in the OS analysis, which exhibited that the association between PhenoAgeAccel and OS was robust and independent of prior treatment exposure (per unit increase: 2.38% risk; 95% CI: 0.7–4.1%; *P* = 0.006; Table S5).

Furthermore, among long-term survivors with early-onset lung cancer in the UK Biobank validation cohort, higher PhenoAgeAccel was also associated with poorer OS (HR = 1.65, 95% CI: 1.06–2.56; *P* = 0.027) (Fig. 5B; Table S6).

## Discussion

The principal challenge in early-onset lung cancer lies in its delayed diagnosis. Numerous studies have shown that such patients are frequently identified at advanced stages, as many seek medical attention only once overt clinical symptoms arise. This reflects the absence of an effective early screening strategy for this subgroup. Our study provides compelling evidence that biological ageing—measured by PhenoAgeAccel using routinely available clinical blood parameters—is closely associated with both the risk and prognosis of early-onset lung cancer. Evidence from our case–control and prognostic cohort analysis with validation of longitudinal UK Biobank data consistently demonstrates that biological age advancement may contribute to the risk and adverse outcomes of early-onset lung cancer. Furthermore, our results suggest that PhenoAgeAccel can help distinguish early-stage lung cancer—particularly from stage II onwards—and may serve as an affordable, accessible, and potentially valuable biomarker for early detection in this population, pending further validation.

Ageing induces structural and functional changes in lung tissue, including reduced regenerative capacity, chronic inflammation, and altered immune responses, thereby creating macro- and microenvironments conducive to carcinogenesis.^32^^,^^33^ Several epigenetic biomarkers have been developed to quantify biological ageing, such as the Klemera–Doubal method, PhenoAge, GrimAge, and telomere length, and are increasingly applied in ageing research.^34^ PhenoAge is based on methylation at CpG sites that regulate the cell cycle, apoptosis, and immune signalling, aligning closely with core carcinogenic pathways. Given their robust predictive capacity and clinical accessibility, PhenoAge and PhenoAgeAccel were utilised in this study to investigate the relationship between biological ageing and early-onset lung cancer. In our research, the absolute values of PhenoAge and chronological age appeared numerically similar in younger patients, leading to a negative mean PhenoAgeAccel. In younger populations, the overall distribution of PhenoAge tends to be younger than chronological age because PhenoAge was originally trained in elder National Health and Nutrition Examination Survey (NHANES) cohorts and validated in middle-to-late adulthood.^30^ This baseline shift has also been reported in UK Biobank analyses, where the mean PhenoAgeAccel values were negative, reflecting cohort-level calibration rather than the absence of biological ageing.^22^^,^^35^

Importantly, although younger individuals tended to show negative PhenoAgeAccel values due to the calibration of the original PhenoAge model in an adult population enriched for middle-to-older ages, this does not imply that PhenoAge lacks interpretability in younger persons. Prior work demonstrates that substantial inter-individual variability in biological ageing is already observable in early adulthood, and such differences meaningfully predict functional decline and disease risk even when absolute biological age estimates are lower than chronological age. Moreover, the residual-based formulation of PhenoAgeAccel was specifically designed to remove the linear effect of chronological age, allowing the metric to represent age-adjusted biological age deviation rather than absolute ageing level. Therefore, although the mean biological age of early-onset lung cancer patients was numerically close to their chronological age, their PhenoAgeAccel values were significantly higher than age-matched controls, indicating that, relative to peers of the same age, these patients exhibited biological age advancement.

Unlike prior studies that did not stratify by age, we provide evidence of the predictive value of PhenoAgeAccel specifically in younger lung cancer patients.^23^, ^24^, ^25^, ^26^, ^27^ The stronger association in this group may reflect heightened susceptibility to environmental and genetic influences, consistent with reported interactions between PhenoAgeAccel and polygenic risk scores.^23^ In contrast, lung cancer in elderly adults may be driven more by cumulative exposures and immunosenescence, potentially explaining the attenuated association in that group.^36^^,^^37^

Our findings align with prior evidence that inflammatory and metabolic markers incorporated into ageing algorithms (*e.g*., CRP, albumin, glucose) contribute to lung cancer development.^38^, ^39^, ^40^ Elevated CRP and white blood cell counts indicate chronic inflammation, which promotes lung cancer progression through DNA damage, creation of a tumour-permissive microenvironment, and increased oxidative stress.^41^ Ageing further skews immune cell composition towards a pro-inflammatory phenotype, increasing populations of myeloid cells such as monocytes and neutrophils that suppress anti-tumour immunity.^42^ These cells release immunosuppressive cytokines such as IL-10 and TGF-β, inhibiting T cell activation and facilitating tumour growth. Chronic inflammation can also induce immune exhaustion, marked by elevated expression of inhibitory receptors such as PD-1, diminishing tumour antigen responsiveness.^43^ Low albumin levels may reflect chronic inflammation or liver dysfunction, both associated with higher lung cancer risk.^44^ A meta-analysis further linked prediabetes with increased lung cancer incidence and mortality, likely mediated by oxidative stress, insulin resistance, and accumulation of advanced glycation end products from hyperglycaemia.^45^ However, our uni- and multivariable logistic regression analyses suggest that these inflammatory and metabolic factors are best interpreted collectively within an ageing framework such as PhenoAge, as individual effect sizes were modest.

In both our prognostic cohort and UK Biobank validation, higher PhenoAgeAccel strongly predicted poorer survival, underscoring its prognostic utility. Even adjusting for relevant confounding variables including prior treatment, PhenoAgeAccel remained an independent and statistically significant predictor of OS. This is consistent with a growing body of work recognising biological age metrics as predictors of adverse cancer outcomes.^46^, ^47^, ^48^ We therefore propose that biological ageing may not be merely a passive correlate of risk but could contribute to tumour progression and diminished host resilience through mechanisms such as systemic inflammation and immune dysregulation. Accordingly, PhenoAgeAccel could serve as a clinically relevant prognostic biomarker to guide personalised treatment and risk management in early-onset lung cancer. However, given that these patients differed substantially in stage, histological subtype, driver gene status, and treatment regimens, further survival-related investigations—such as analyses of PFS or subgroup OS—are warranted in appropriately stratified populations. The further stratified analyses by mutation status suggests that, among patients without known driver mutations, PhenoAgeAccel may be associated with a stronger adverse prognostic effect than among patients harbouring known driver mutations, whether EGFR or other oncogenic alterations Nonetheless, these subgroup findings should be considered exploratory due to small sample sizes and wide confidence intervals.

In summary, our results indicate that biological ageing, reflected by PhenoAgeAccel, offers a lens for understanding the aetiology and progression of early-onset lung cancer in younger adults. Although the limited number of early-onset lung cancer cases in the UK Biobank validation constrains statistical power, the consistency of associations across independent designs and populations supports the potential relevance of PhenoAgeAccel as a marker integrating with other factors for risk stratification. Future research should aim to validate these findings in larger, ethnically diverse, multi-centre prospective cohorts, incorporating variations in environmental exposure and clinical presentation. Mechanistic studies employing transcriptomic, epigenetic, and proteomic profiling of tumours and adjacent tissues could elucidate the biological pathways linking biological ageing to early-onset lung cancer, such as dysregulated inflammation, oxidative stress, and impaired DNA repair. Moreover, targeting ageing-related pathways through anti-inflammatory therapies, metabolic modulation, or senolytic agents may offer strategies for improving patient outcomes. Understanding how such interventions interact with the unique biology of early-onset lung cancer could pave the way for innovative, age-tailored treatments.

Despite its strengths, our study has limitations. First, the case–control study involved only a Chinese population. Although our power analysis confirmed adequate sensitivity for key effect sizes, replication in larger samples is required. The UK Biobank validation cohort contained relatively few younger individuals who developed lung cancer, and biomarker measurements occurred at varying times before diagnosis, limiting statistical power and may reduce the robustness and generalisability of the validation results. Second, while PhenoAgeAccel showed promise in identifying early-stage lung cancer from stage II onwards, the small number of early-stage cases limited statistical power. Larger early-stage cohorts are needed for robust validation. Third, PhenoAgeAccel was derived from blood-based biomarkers measured at a single time point. Biological ageing is a dynamic process, and single measurements may not fully capture temporal changes in physiological ageing or the influence of transient factors such as inflammation, metabolic stress, or acute illness. Moreover, unmeasured confounders—such as early-life health status, socioeconomic context, or cohort effects—could influence both biological ageing and cancer risk, potentially biasing effect estimates. Longitudinal studies with repeated biomarker assessments will be essential to clarify causality and temporal trajectories. Furthermore, the moderate AUC suggests that although PhenoAgeAccel provides useful prognostic and risk stratification information, it may not be sufficient as a standalone diagnostic or predictive biomarker. Nevertheless, its simplicity and reliance on routinely available blood chemistry parameters make it an attractive and accessible tool for preliminary risk screening. In this context, PhenoAgeAccel could help identify individuals at elevated risk for early-onset lung cancer, thereby improving the efficiency of downstream clinical screening like LDCT and facilitating targeted prevention strategies. Finally, observed differences in the association between PhenoAgeAccel and lung cancer across age groups may be confounded by unmeasured factors such as comorbidities, treatment variations, and baseline health, which could partly explain the observed heterogeneity. Stratified analyses in larger datasets and mechanistic studies are needed to disentangle these interrelated factors.

In conclusion, this study identifies PhenoAgeAccel as a significant contributor to the risk and prognosis of early-onset lung cancer. Consistent findings across populations support PhenoAgeAccel as a feasible biomarker for risk stratification in early-onset lung cancer. Furthermore, its simple calculation allows for integration with established risk factors, such as family history and smoking, to help identify high-risk individuals. Additionally, PhenoAgeAccel may help inform preventive or adjunctive anti-ageing interventions aimed at improving long-term outcomes.

## Contributors

Concept and Design: Jia Zhong, Jie Wang. Data acquisition: Ruolin Gao, Yuxuan Liao, Zhijian Xu, Xiaowei Zhao, Qinxiang Guo, Zhijie Wang, Jianchun Duan, Rui Wan, Jiachen Xu, Kailun Fei, Boyang Sun, Wei Zhuang, Yanhui Cheng, and Hua Bai. The underlying data accessing and verifying: Ruolin Gao, Yuxuan Liao, Jia Zhong, and Jie Wang. Data analysis and interpretation: Ruolin Gao, Yuxuan Liao, Qiwen Zheng, and Zhijian Xu. Manuscript drafting: Ruolin Gao, Yuxuan Liao. Critical revision: Qiwen Zheng, Jia Zhong, and Kailun Fei. Funding and supervision: Jia Zhong, Jie Wang. All authors have read and approved the final manuscript.

## Data sharing statement

The de-identified clinical data generated in this study are available from the corresponding authors upon reasonable request. Researchers with a justified academic purpose may obtain access by submitting a brief proposal and, if applicable, proof of ethical approval. Data will be shared after publication and will remain available for five years. Requests should be sent to corresponding authors’ email (zhong-jia817@163.com or zlhuxi@163.com). A data access agreement will be required prior to data release. The source code of the analysis can be obtained via https://github.com/liaoyuxuan0806-glitch/Young-lung-cancer-and-accelerated-biological-ageing.

## Declaration of interests

All the authors declare no conflicts of interest.
