# Supplementary material for: Phenotypic age acceleration and early-onset lung cancer: a case–control and prognostic cohort study involving multiple clinical centres with validation in the UK Biobank
Source: eBioMedicine. 2026 Feb 10;125:106162. doi: 10.1016/j.ebiom.2026.106162 (PMC12914819; doi:10.1016/j.ebiom.2026.106162)
Supplement: Supplementary Figures and Tables [file mmc1.docx]

**Supplementary Materials**

**Supplementary Method**

**Sample Size Calculation and Power Analysis**

The sample size for this study was estimated based on the expected effect size of PhenoAgeAccel. According to preliminary research findings and clinical significance, we anticipated a between-group difference of 3 units with an expected standard deviation of 10 units.^1^ Using a two-sided test with a significance level ($\alpha$) of 0.05 and statistical power (1-$\beta$) of 0.80, the sample size was calculated.

The standard sample size calculation formula was applied:

$$n=\frac{{(Z_{\alpha/2}+Z_{\beta})}^{2}\sigma^{2}}{\varepsilon^{2}}$$

where n represents the required sample size per group, Z values correspond to the critical values for the significance level and statistical power, $\sigma$ is the standard deviation, and $\varepsilon$ is the expected between-group difference.

$n=$[(1.96 + 0.84)²×10²]/3² = 87.11 per group

Based on the calculation, a minimum of 87.11 (rounded up to 88) subjects per group was required.

To validate our sample size calculation, we constructed a reference table showing the relationship between expected differences and standard deviations across various scenarios (Table 1).

Table 1. Sample Size Requirements (80% Power, Two-tailed $\alpha$= 0.05)

| Expected difference | Expected standard deviation | | | | | | | | | |
| --- | --- | --- | --- | --- | --- | --- | --- | --- | --- | --- |
|  | 1 | 2 | 3 | 4 | 5 | 6 | 7 | 8 | 9 | 10 |
| 1 | 8 | 32 | 71 | 126 | 197 | 284 | 386 | 505 | 641 | 794 |
| 2 | 2 | 8 | 18 | 32 | 49 | 71 | 97 | 126 | 160 | 198 |
| 3 | 1 | 4 | 8 | 14 | 22 | 32 | 43 | 56 | 71 | 88 |
| 4 | 1 | 2 | 5 | 8 | 13 | 18 | 24 | 32 | 40 | 50 |
| 5 | 1 | 2 | 3 | 6 | 8 | 12 | 16 | 20 | 26 | 32 |
| 6 | 1 | 2 | 3 | 4 | 6 | 8 | 11 | 14 | 18 | 22 |

*All values represent the minimum required sample size (rounded up)

Post-hoc power calculation

Post-hoc power analysis was conducted to evaluate the statistical power of our study based on the observed effect sizes. With a total sample size of 444 young participants divided into 2 equal groups (222 participants per group, young lung cancer patients and healthy young adults), power calculations were performed using the following formula:

$$Power=1-\beta=\Phi(Z_{\mathrm{observed}}-Z_{\alpha/2})$$

Where:

- $Z_{\mathrm{observed}}$ represents the standardized effect size

- $Z_{\alpha/2}$ equals 1.96 for α=0.05 (two-tailed)

- Φ denotes the standard normal cumulative distribution function

For two-sample comparisons with 222 subjects per group (total N=444):

| Effect Size (Cohen's d) | Power (1-β) |
| --- | --- |
| 0.1 (very small) | 55.3% |
| 0.2 (small) | 97.8% |
| 0.3 (small-medium) | >99.9% |
| 0.5 (medium) | >99.9% |
| 0.8 (large) | >99.9% |

**PhenoAge Calculation**

PhenoAge is a validated biological age biomarker developed by Levine et al. using data from the third National Health and Nutrition Examination Survey (NHANES III), a nationally-representative sample of 9,926 US adults with >23 years of mortality follow-up.^2^ A Cox penalized regression model—where the hazard of mortality was regressed on forty-two clinical markers and chronological age—was used to select variables for inclusion in phenotypic age score. Based on 10-fold cross-validation, nine biomarkers and chronological age were then combined in a phenotypic age estimate (in units of years) as detailed below:

$$PhenoAge=141.50+{{\ln(-0.00553\times-1.51714\times e^{xb})}^{0.0076927}}^{0.09165}$$

where

$xb=-19.907-0.0336\times albumin+0.0095\times creatinine+0.1953\times glucose+0.0954\times ln(CRP)-0.0120\times lymphocyte percentage+0.0268\times mean corpuscular volume+0.3306\times RDW+0.00188\times alkaline phosphatase+0.0554\times white blood cell count+0.0804\times age$

PhenoAge has been validated using data from NHANES IV (n=6,209), demonstrating excellent correlation with chronological age (r=0.94) and robust associations with mortality outcomes. Specifically, each 1-year increase in PhenoAge was associated with a 9% increased risk of all-cause mortality (HR=1.09, p=3.8×10^⁻49^), a 9% increased risk of aging-related disease mortality (HR=1.09, p=4.5×10^⁻34^), and significant increases in cause-specific mortality risks, including a 7% increased risk of cancer mortality (HR=1.07, p=7.9×10^-10^). The biomarker has subsequently been applied in large-scale population studies, including the UK Biobank cohort.^3^

We calculated PhenoAge acceleration (PhenoAgeAccel) as the residual from regressing PhenoAge on chronological age, representing how much older (positive values) or younger (negative values) an individual's biological age is compared to their chronological age. This approach is justified given PhenoAge's demonstrated validity across several large-scale studies and its strong predictive validity for health outcomes in diverse populations.

**Reference**

1. Kuo CL, Pilling LC, Liu Z, Atkins JL, Levine ME. Genetic associations for two biological age measures point to distinct aging phenotypes. Aging Cell. 2021;20(6):e13376. doi:10.1111/acel.13376
2. Levine ME, Lu AT, Quach A, et al. An epigenetic biomarker of aging for lifespan and healthspan. Aging (Albany NY). 2018;10(4):573-591. doi:10.18632/aging.101414
3. Kuo CL, Pilling LC, Atkins JL, et al. Biological Aging Predicts Vulnerability to COVID-19 Severity in UK Biobank Participants. J Gerontol A Biol Sci Med Sci. 2021;76(8):e133-e141. doi:10.1093/gerona/glab060

**Supplementary Figures**


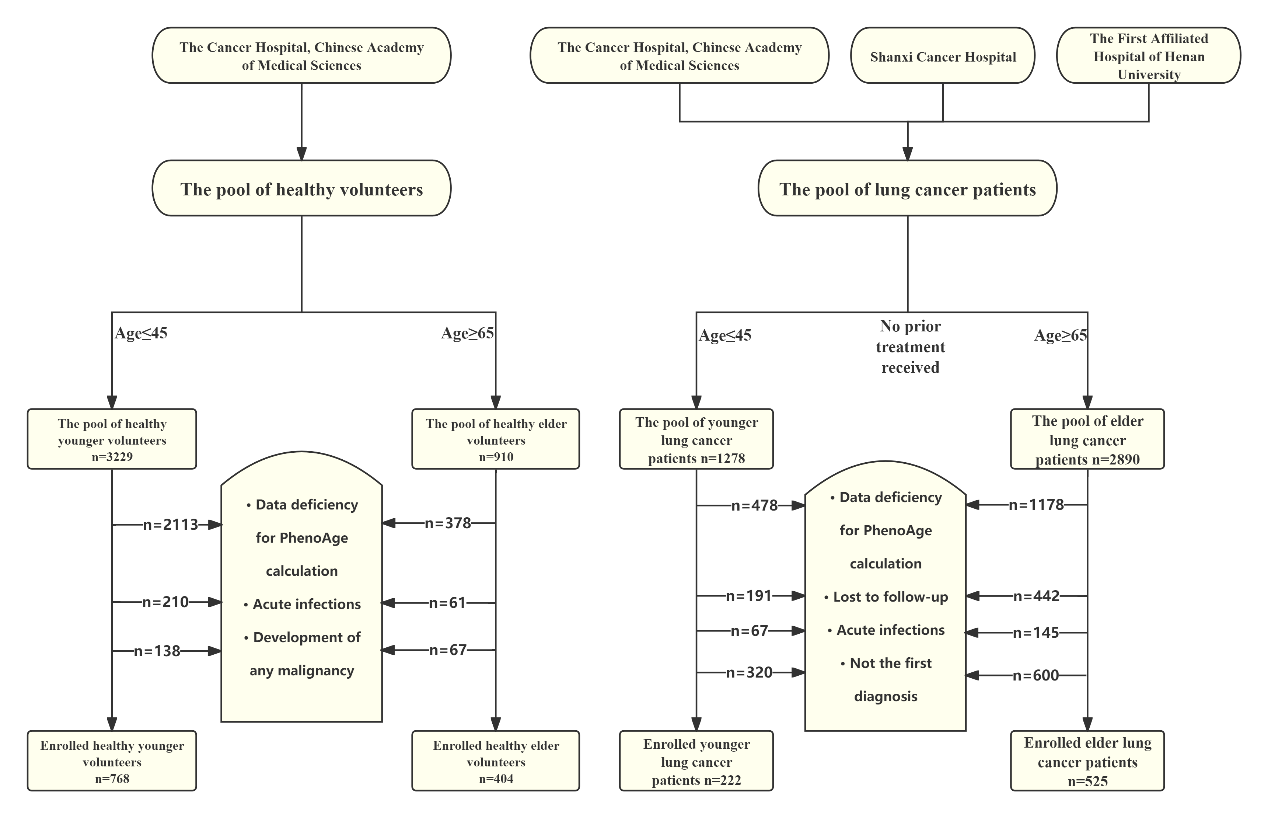


**Figure S1 The specific inclusion and exclusion flowchart of the study.**


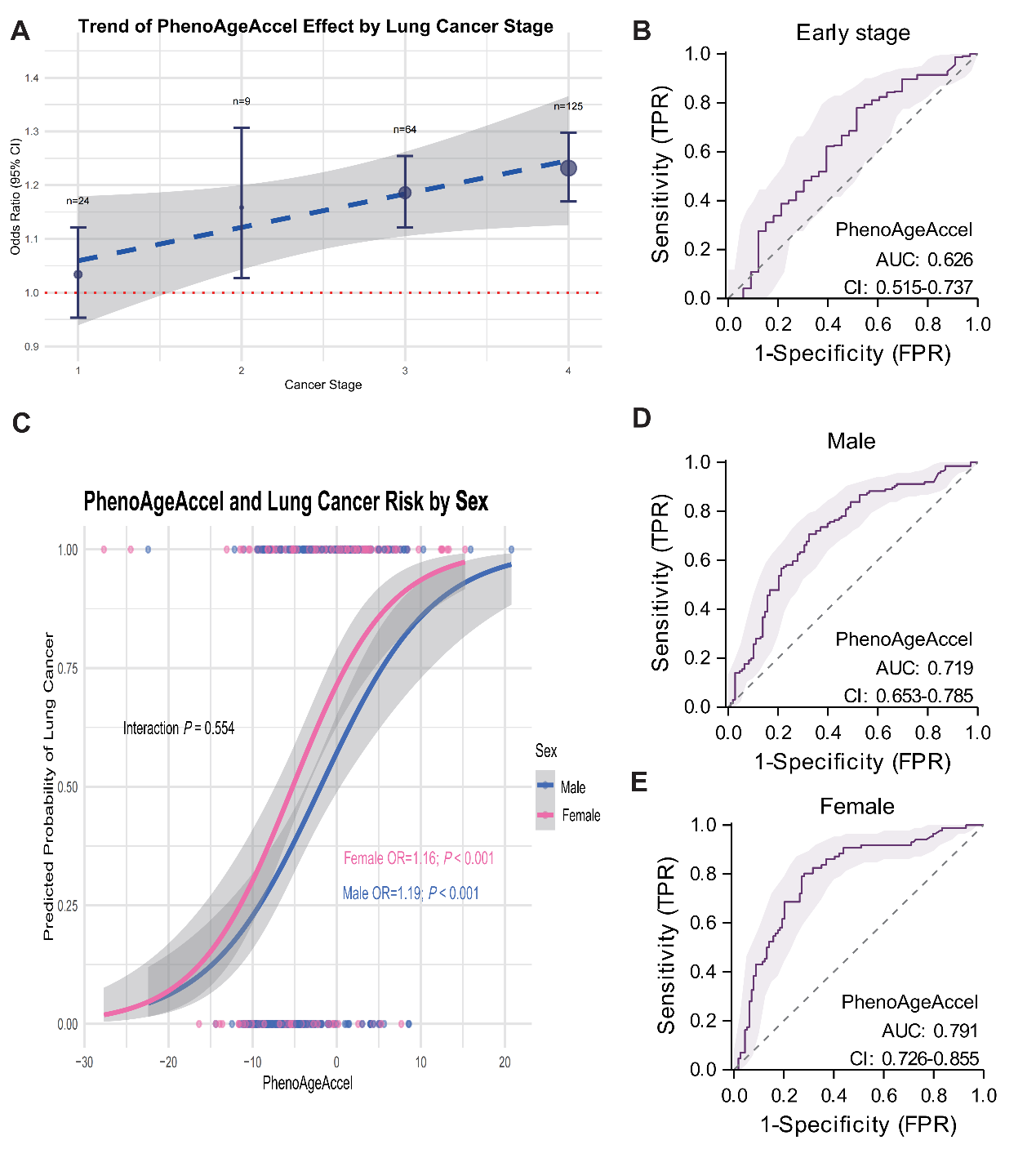


**Figure S2 Stratified analyses of the association between accelerated biological aging and early-onset lung cancer risk by cancer stage and sex.** (A) Trend analysis showing the effect of phenotypic age acceleration (PhenoAgeAccel) on early-onset lung cancer risk stratified by cancer stage. The odds ratios (ORs) with 95% confidence intervals (CIs) were plotted for each stage (Stage I to IV), indicating an increasing trend in risk with higher stages of lung cancer (dashed blue line), suggesting a stage-dependent effect of PhenoAgeAccel. (B) Receiver operating characteristic (ROC) curve assessing the predictive performance of PhenoAgeAccel in younger patients with early-stage (Stage I to II) lung cancer, with the area under the curve (AUC) of 0.626 (95% CI: 0.515–0.737). (C) Stratified analysis by sex showing the predicted probability of early-onset lung cancer across different levels of PhenoAgeAccel. Both females (pink line) and males (blue line) exhibit a significant positive association between PhenoAgeAccel and lung cancer risk (Female OR = 1.16, P < 0.001; Male OR = 1.19, P < 0.001), with no significant interaction by sex (P for interaction = 0.554). (D) ROC curve evaluating the predictive performance of PhenoAgeAccel for early-onset lung cancer in males, with the AUC of 0.719 (95% CI: 0.653–0.785). (E) ROC curve evaluating the predictive performance of PhenoAgeAccel for early-onset lung cancer in females, with the AUC of 0.791 (95% CI: 0.726–0.855).


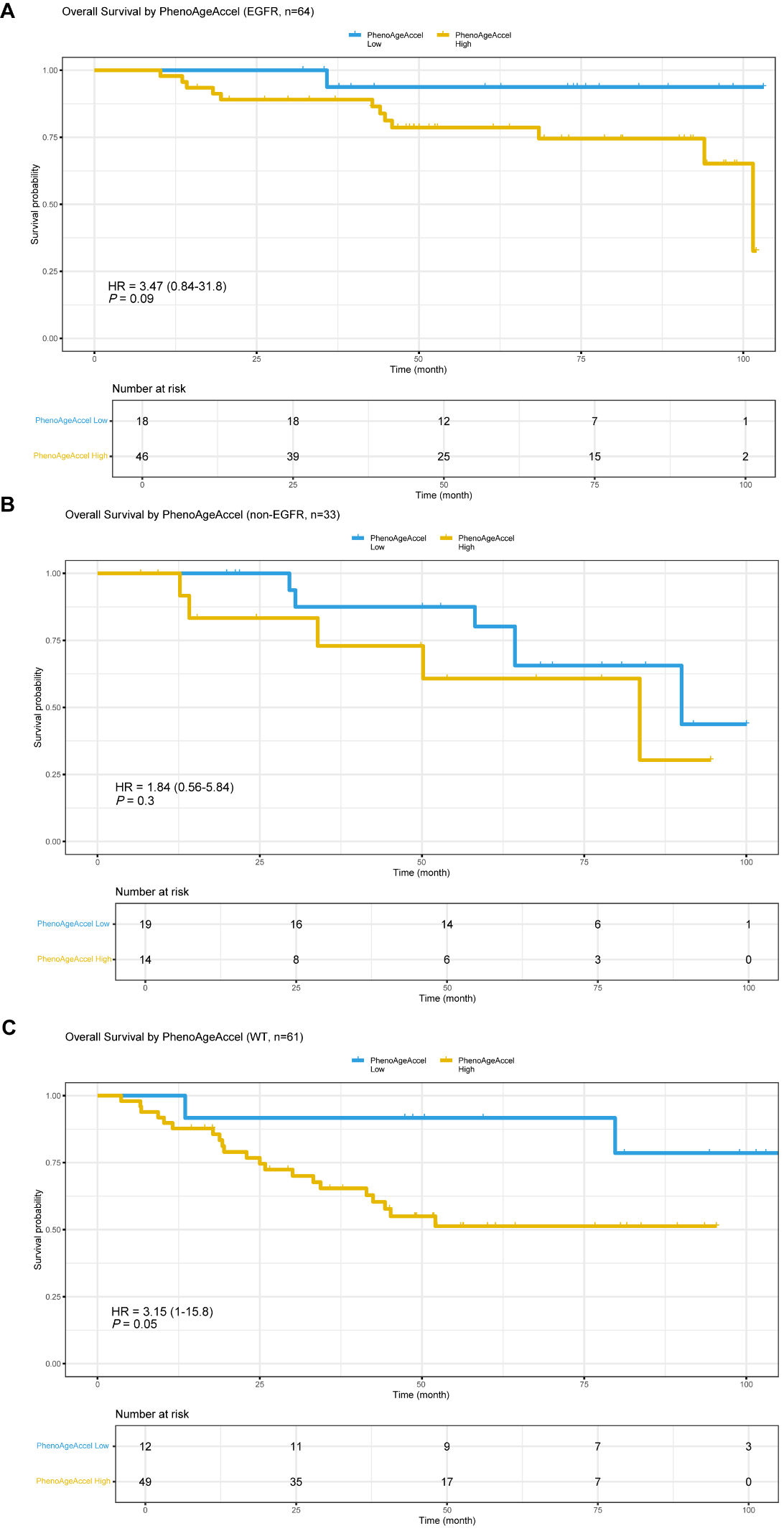


**Figure S3 Overall survival of early-onset lung cancer patients stratified by PhenoAgeAccel levels in different genetic mutation subgroups**. Hazard ratios (HRs) were estimated using univariate Cox proportional hazards models, with the number at risk shown below each plot. The cutoff for dividing high and low PhenoAgeAccel groups was based on the optimal P value calculation. (A) In EGFR-mutated patients (n = 64), those with high PhenoAgeAccel showed a trend toward poorer OS compared to the low PhenoAgeAccel group (HR = 3.47, 95% CI: 0.84–31.8; P = 0.09). The cutoff was -5.37. (B) In non-EGFR-mutated patients (n = 33), a similar pattern was observed, although not statistically significant (HR = 1.84, 95% CI: 0.56–5.84; P = 0.3). The cutoff was 0.77. (C) Among wild-type (WT) patients (n = 61), high PhenoAgeAccel was significantly associated with poorer OS (HR = 3.15, 95% CI: 1–15.8; P = 0.05). The cutoff was -7.6.

| **Table S1 Characteristics of the propensity score–matched younger and elder adult patients with lung cancer** | | | | | | | | |
| --- | --- | --- | --- | --- | --- | --- | --- | --- |
|  | **Before matching** | | |  | **After matching** | | |  |
|  | **Younger adults with lung cancer**  **N=222** | **Elder adults with lung cancer**  **N=525** | ***P*** | **SMD** | **Younger adults with lung cancer**  **N=222** | **Elder adults with lung cancer**  **N=222** | ***P*** | **SMD** |
| **Sex** |  |  | <0.001 | 0.505 |  |  | 0.296 | 0.108 |
| **Male** | 102 (45.9) | 368 (70.1) |  |  | 102 (45.9) | 114 (51.4) |  |  |
| **Female** | 120 (54.1) | 157 (29.9) |  |  | 120 (54.1) | 108 (48.6) |  |  |
| **Smoking History** |  |  | <0.001 | 0.684 |  |  | 0.281 | 0.112 |
| **No** | 145 (65.3) | 173 (33.0) |  |  | 145 (65.3) | 145 (65.3) |  |  |
| **Yes** | 77 (34.7) | 352 (67.0) |  |  | 77 (34.7) | 77 (34.7) |  |  |
| **Family History** |  |  | 0.734 | 0.034 |  |  | 1.000 | 0.010 |
| **No** | 162 (73.0) | 375 (71.4) |  |  | 162 (73.0) | 162 (73.0) |  |  |
| **Yes** | 60 (27.0) | 150 (28.6) |  |  | 60 (27.0) | 60 (27.0) |  |  |
| **Histology** |  |  | <0.001 | 0.564 |  |  | 0.497 | 0.147 |
| **Adenocarcinoma** | 175 (78.8) | 283 (53.9) |  |  | 175 (78.8) | 166 (74.8) |  |  |
| **Squamous carcinoma** | 20 (9.0) | 125 (23.8) |  |  | 20 (9.0) | 22 (9.9) |  |  |
| **Small cell lung cancer** | 18 (8.1) | 90 (17.1) |  |  | 18 (8.1) | 27 (12.2) |  |  |
| **Others** | 9 (4.1) | 27 (5.1) |  |  | 9 (4.1) | 7 (3.2) |  |  |
| **ECOG PS Score** |  |  | ＜0.001 | 0.479 |  |  | 0.437 | 0.122 |
| **0** | 155 (69.8) | 252 (48.0) |  |  | 155 (69.8) | 144 (64.9) |  |  |
| **1** | 65 (29.3) | 249 (47.4) |  |  | 65 (29.3) | 74 (33.3) |  |  |
| **2** | 2 (0.9) | 21 (4.0) |  |  | 2 (0.9) | 4 (1.8) |  |  |
| **3** | 0 (0.0) | 3 (0.6) |  |  | 0 (0.0) | 0 |  |  |
| **Stage** |  |  | 0.002 | 0.331 |  |  | 0.852 | 0.084 |
| **I** | 24 (10.8) | 44 (8.4) |  |  | 24 (10.8) | 20 (9.0) |  |  |
| **II** | 9 (4.1) | 59 (11.2) |  |  | 9 (4.1) | 11 (5.0) |  |  |
| **III** | 64 (28.8) | 182 (34.7) |  |  | 64 (28.8) | 69 (31.1) |  |  |
| **IV** | 125 (56.3) | 240 (45.7) |  |  | 125 (56.3) | 122 (55.0) |  |  |
| **T** |  |  | 0.026 | 0.239 |  |  | 0.859 | 0.083 |
| **T1** | 65 (29.3) | 102 (19.4) |  |  | 65 (29.3) | 61 (27.5) |  |  |
| **T2** | 70 (31.5) | 196 (37.3) |  |  | 70 (31.5) | 65 (29.3) |  |  |
| **T3** | 33 (14.9) | 95 (18.1) |  |  | 33 (14.9) | 37 (16.7) |  |  |
| **T4** | 54 (24.3) | 132 (25.1) |  |  | 54 (24.3) | 59 (26.6) |  |  |
| **N** |  |  | 0.454 | 0.131 |  |  | 0.815 | 0.092 |
| **N0** | 36 (16.2) | 99 (18.9) |  |  | 36 (16.2) | 32 (14.4) |  |  |
| **N1** | 20 (9.0) | 61 (11.6) |  |  | 20 (9.0) | 25 (11.3) |  |  |
| **N2** | 86 (38.7) | 178 (33.9) |  |  | 86 (38.7) | 89 (40.1) |  |  |
| **N3** | 80 (36.0) | 187 (35.6) |  |  | 80 (36.0) | 76 (34.2) |  |  |
| **M** |  |  | 0.005 | 0.234 |  |  | 0.568 | 0.063 |
| **M0** | 99 (44.6) | 295 (56.2) |  |  | 99 (44.6) | 106 (47.7) |  |  |
| **M1** | 123 (55.4) | 230 (43.8) |  |  | 123 (55.4) | 116 (52.3) |  |  |
| **Laterality** |  |  | 0.665 | 0.072 |  |  | 0.981 | 0.018 |
| **Others** | 2 (0.9) | 5 (1.0) |  |  | 2 (0.9) | 2 (0.9) |  |  |
| **Right** | 131 (59.0) | 291 (55.4) |  |  | 131 (59.0) | 133 (59.9) |  |  |
| **Left** | 89 (40.1) | 229 (43.6) |  |  | 89 (40.1) | 87 (39.2) |  |  |
| **Medical center** |  |  |  |  |  |  | 0.650 | 0.088 |
| **Center 1** | 180 (81.1) | 444 (84.6) | 0.262 | 0.126 | 180 (81.1) | 182 (82.0) |  |  |
| **Center 2** | 20 (9.0) | 47 (9.0) |  |  | 20 (9.0) | 23 (10.4) |  |  |
| **Center 3** | 22 (9.9) | 34 (6.5) |  |  | 22 (9.9) | 17 (7.7) |  |  |
| Categorical variables were presented as n (%). For comparison between groups of categorical data, we used the Fisher exact test for expected frequencies of <5, otherwise, we used the Chi-squared test.  Propensity score matching (PSM) was used to balance sex, smoking history, family history, histology, ECOG PS score, tumor TNM stage, laterality and medical center. Center 1: the Cancer Hospital, Chinese Academy of Medical Sciences; Center 2: the Shanxi Cancer Hospital; Center 3: the First Affiliated Hospital of Henan University | | | | | | | |  |

| **Table S2 Characteristics of propensity score–matched younger adult lung cancer patients and healthy volunteers** | | | | | | | |  |
| --- | --- | --- | --- | --- | --- | --- | --- | --- |
|  | **Before matching** | | |  | **After matching** | | |  |
|  | **Younger adults with lung cancer N=222** | **Healthy younger volunteers**  **N=768** | ***P*** | **SMD** | **Younger adults with lung cancer**  **N=222** | **Healthy younger volunteers**  **N=222** | ***P*** | **SMD** |
| **Age** | 40±5.15 | 40±6.71 | ＜0.001 | 1.136 | 40±5.15 | 40±5.10 | 1 | 0.180 |
| **Sex** |  |  |  |  |  |  |  |  |
| **Male** | 102 (45.9) | 454 (59.0) | 0.001 | 0.264 | 102 (45.9) | 100 (45) | 0.924 | 0.018 |
| **Female** | 120 (54.1) | 315 (41.0) |  |  | 120 (54.1) | 122 (55) |  |  |
| **Smoking History** |  |  |  |  |  |  |  |  |
| **No** | 145 (65.3) | 464 (60.3) | 0.182 | 0.104 | 145 (65.3) | 144 (64.5) | 0.909 | 0.017 |
| **Yes** | 77 (34.7) | 304 (39.7) |  |  | 77 (34.7) | 78 (35.5) |  |  |
| **Family History** |  |  |  |  |  |  |  |  |
| **No** | 162 (73.0) | 480 (62.4) | 0.04 | 0.228 | 162 (73.0) | 159 (71.6) | 0.747 | 0.031 |
| **Yes** | 60 (27.0) | 288 (37.6) |  |  | 60 (27.0) | 63 (28.4) |  |  |
| Categorical variables were presented as n (%). Chi-squared test was used for the statistical analysis of categorical variables.  Propensity score matching (PSM) was used to balance age, sex, smoking history, and family history. | | | | | | | | |

| **Table S3 Characteristics of propensity score-matched elder lung cancer patients and health volunteers** | | | | | | | |  |
| --- | --- | --- | --- | --- | --- | --- | --- | --- |
|  | **Before matching** | | |  | **After matching** | | |  |
|  | **Elder adults with lung cancer**  **N=222** | **Healthy elder volunteers**  **N=404** | ***P*** | **SMD** | **Elder adults with lung cancer N=222** | **Healthy elder volunteers**  **N=222** | ***P*** | **SMD** |
| **Age** | 68±3.92 | 69±2.70 | ＜0.001 | 0.301 | 68±3.92 | 69±3.34 | 0.886 | 0.274 |
| **Sex** |  |  | 0.566 | 0.046 |  |  | 0.849 | 0.027 |
| **Male** | 114 (51.4) | 199 (49.1) |  |  | 114 (51.4) | 117 (52.7) |  |  |
| **Female** | 108 (48.6) | 205 (50.9) |  |  | 108 (48.6) | 105 (47.3) |  |  |
| **Smoking History** |  |  | 0.053 | 0.161 |  |  | 0.291 | 0.103 |
| **No** | 133 (59.9) | 273 (67.8) |  |  | 133 (59.9) | 144 (64.9) |  |  |
| **Yes** | 89 (40.1) | 131 (32.2) |  |  | 89 (40.1) | 78 (35.1) |  |  |
| **Family History** |  |  | 0.281 | 0.096 |  |  | 0.748 | 0.029 |
| **No** | 163 (73.4) | 279 (69.1) |  |  | 163 (73.4) | 160 (72.1) |  |  |
| **Yes** | 59 (26.6) | 125 (30.9) |  |  | 59 (26.6) | 62 (27.9) |  |  |
| Categorical variables were presented as n (%). Chi-squared test was used for the statistical analysis of categorical variables.  Propensity score matching (PSM) was used to balance age, sex, smoking history, and family history. | | | | | | | | |

| **Table S4 Multivariate Cox proportional hazards analysis for overall survival of early-onset lung cancer patients in the** **prognostic cohort study** | | | |
| --- | --- | --- | --- |
|  | **Hazard ratio (HR)** | **95% confidence intervals (CI)** | **P value** |
| **PhenoAgeAccel group** |  |  |  |
| **Low** | Ref. |  |  |
| **High** | 2.17 | 1.20-3.93 | **0.010** |
| **Chronological age** | 1.05 | 0.99-1.10 | 0.101 |
| **Sex** |  |  |  |
| **male** | Ref. |  |  |
| **female** | 0.88 | 0.48-1.63 | 0.696 |
| **Smoking history** |  |  |  |
| **No** | Ref. |  |  |
| **Yes** | 1.44 | 0.75-2.76 | 0.269 |
| **Family history** |  |  |  |
| **No** | Ref. |  |  |
| **Yes** | 0.71 | 0.38-1.31 | 0.270 |
| **Histology** |  |  |  |
| **NSCLC** | Ref. |  |  |
| **SCLC** | 1.68 | 0.69-4.06 | 0.253 |
| **Stage** |  |  |  |
| **Early** | Ref. |  |  |
| **Late** | 3.87 | 1.19-12.60 | **0.025** |
| **Medical center** |  |  |  |
| **Center 1** | Ref. |  |  |
| **Center 2** | 0.37 | 0.11-1.23 | 0.105 |
| **Center 3** | 0.24 | 0.06-1.01 | 0.051 |
| PhenoAgeAccel was defined as the residual from a linear regression model of PhenoAge on chronological age. The high PhenoAgeAccel group was defined as those with a value greater than or equal to -3.42, while the low PhenoAgeAccel group included those with a value less than -3.42. A cutoff of -3.42 was determined based on the optimal P value calculation.  Multivariate Cox proportional hazards analysis was used to compare the overall survival of early-onset lung cancer patients by PhenoAgeAccel group. PhenoAgeAccel, PhenoAge acceleration. NSCLC, non-small cell lung cancer. SCLC, small cell lung cancer. Early stage including stage I and II; late stage including stage III and IV. Center 1: the Cancer Hospital, Chinese Academy of Medical Sciences; Center 2: the Shanxi Cancer Hospital; Center 3: the First Affiliated Hospital of Henan University. | | | |

| **Table S5 Multivariate Cox proportional hazards analysis for overall survival of early-onset lung cancer patients in the prognostic cohort study incorporating prior treatments** | | | |
| --- | --- | --- | --- |
|  | **Hazard ratio (HR)** | **95% confidence intervals (CI)** | **P value** |
| **PhenoAgeAccel (continuous)** | 1.02 | 1.01-1.04 | **0.006** |
| **Chronological age** | 1.04 | 0.98-1.09 | 0.115 |
| **Immunotherapy** |  |  |  |
| **No** | Ref. |  |  |
| **Yes** | 0.91 | 0.63-1.30 | 0.592 |
| **Radiotherapy** |  |  |  |
| **No** | Ref. |  |  |
| **Yes** | 1.07 | 0.77-1.49 | 0.677 |
| **TKIs** |  |  |  |
| **No** | Ref. |  |  |
| **Yes** | 1.02 | 0.76-1.38 | 0.893 |
| **Surgery** |  |  |  |
| **No** | Ref. |  |  |
| **Yes** | 0.75 | 0.44-1.30 | 0.308 |
| **Chemotherapy** |  |  |  |
| **No** | Ref. |  |  |
| **Yes** | 0.97 | 0.79-1.21 | 0.806 |
| **Sex** |  |  |  |
| **male** | Ref. |  |  |
| **female** | 0.90 | 0.49-1.67 | 0.712 |
| **Smoking history** |  |  |  |
| **No** | Ref. |  |  |
| **Yes** | 1.39 | 0.73-2.64 | 0.285 |
| **Family history** |  |  |  |
| **No** | Ref. |  |  |
| **Yes** | 0.74 | 0.39-1.36 | 0.283 |
| **Histology** |  |  |  |
| **NSCLC** | Ref. |  |  |
| **SCLC** | 1.07 | 0.73-1.56 | 0.743 |
| **Stage** |  |  |  |
| **Early** | Ref. |  |  |
| **Late** | 2.00 | 1.30-3.10 | **0.002** |
| **Driver gene mutations** |  |  |  |
| **EGFR** | Ref. |  |  |
| **Non-EGFR** | 1.54 | 1.14-2.09 | **0.005** |
| **WT** | 1.73 | 1.31-2.30 | **<0.001** |
| **Untested** | 2.08 | 1.52-2.84 | **<0.001** |
| **ECOG PS** |  |  |  |
| **0** | Ref. |  |  |
| **≥1** | 2.06 | 1.66-2.56 | **<0.001** |
| PhenoAgeAccel was defined as the residual from a linear regression model of PhenoAge on chronological age.  PhenoAgeAccel, PhenoAge acceleration. TKI, tyrosine kinase inhibitor. NSCLC, non-small cell lung cancer; SCLC, small cell lung cancer. Early stage including stage I and II; late stage including stage III and IV. Non-EGFR driver gene mutations including ERBB2, KRAS, RET, ROS1. ECOG PS, Eastern Cooperative Oncology Group performance status. | | | |

| **Table S6 Multivariate Cox proportional hazards analysis for overall survival of early-onset lung cancer survivors in the UK Biobank validation** | | | |
| --- | --- | --- | --- |
|  | **Hazard ratio (HR)** | **95% confidence intervals (CI)** | **P value** |
| **PhenoAgeAccel group** |  |  |  |
| **Low** | Ref. |  |  |
| **High** | 1.65 | 1.06-2.56 | **0.027** |
| **Chronological age** | 1.10 | 1.06-1.14 | **<0.001** |
| **Sex** |  |  |  |
| **male** | Ref. |  |  |
| **female** | 1.55 | 0.95-2.55 | 0.083 |
| **Smoking history** |  |  |  |
| **No** | Ref. |  |  |
| **Yes** | 0.90 | 0.45-1.81 | 0.770 |
| **Family history** |  |  |  |
| **No** | Ref. |  |  |
| **Yes** | 0.97 | 0.62-1.51 | 0.899 |
| **Race** |  |  |  |
| **Non-White** | Ref. |  |  |
| **White** | 1.86 | 0.85-4.06 | 0.123 |
| **Townsend** | 1.07 | 1.01-1.13 | **0.020** |
| **BMI** | 1.01 | 0.97-1.04 | 0.716 |
| PhenoAgeAccel was defined as the residual from a linear regression model of PhenoAge on chronological age. The high PhenoAgeAccel group was defined as those with a value greater than or equal to -6.05, while the low PhenoAgeAccel group included those with a value less than -6.05. A cutoff of -6.05 was determined based on the optimal P value calculation.  Multivariate Cox proportional hazards analysis was used to compare the overall survival of early-onset lung cancer patients by PhenoAgeAccel group. PhenoAgeAccel, PhenoAge acceleration. | | | |
